# Correction: Ethical challenges of using remote monitoring technologies for clinical research: A case study of the role of local research ethics committees in the RADAR-AD study

**DOI:** 10.1371/journal.pone.0294797

**Published:** 2023-11-16

**Authors:** Marijn Muurling, Anna M. G. Pasmooij, Ivan Koychev, Dora Roik, Lutz Froelich, Emilia Schwertner, Dorota Religa, Carla Abdelnour, Mercè Boada, Monica Almici, Samantha Galluzzi, Sandra Cardoso, Alexandre de Mendonça, Andrew P. Owens, Sajini Kuruppu, Martha Therese Gjestsen, Ioulietta Lazarou, Mara Gkioka, Magda Tsolaki, Ana Diaz, Dianne Gove, Pieter Jelle Visser, Dag Aarsland, Federica Lucivero, Casper de Boer, the RADAR-AD Consortium

There are errors in the Funding section. The correct Funding statement is: The RADAR-AD project has received funding from the Innovative Medicines Initiative 2 Joint Undertaking under grant agreement No 806999. This Joint Undertaking receives support from the European Union’s Horizon 2020 research and innovation programme and EFPIA and Software AG. See https://www.imi.europa.eu/ for more details. This communication reflects the views of the RADAR-AD consortium and neither IMI nor the European Union and EFPIA are liable for any use that may be made of the information contained herein. Research of Alzheimer center Amsterdam is part of the neurodegeneration research program of Amsterdam Neuroscience. Alzheimer Center Amsterdam is supported by Stichting Alzheimer Nederland and Stichting Steun Alzheimercentrum Amsterdam. IK declares support for this work through the National Institute of Health Research (personal award and Oxford Health Biomedical Research Centre) and the Medical Research Council (Dementias Platform UK grant). CA’s postdoctoral fellowship is funded by the Susan and Charles Berghoff Foundation. SG declares support for this work through the Italian Ministry of Health (Ricerca Corrente). The funders had no role in study design, data collection and analysis, decision to publish, or preparation of the manuscript.
