# Supplementary material for: Ethical challenges of using remote monitoring technologies for clinical research: A case study of the role of local research ethics committees in the RADAR-AD study
Source: PLoS One. 2023 Jul 7;18(7):e0285807. doi: 10.1371/journal.pone.0285807 (PMC10328231; doi:10.1371/journal.pone.0285807)
Supplement: S1 Table — (DOCX) [file pone.0285807.s001.docx]

# Supporting information file

*Table S1 – Details of the documents reviewed for this study and review processes in each site*

| **Site** | **Documents reviewed**  (Correspondence with…) | **Review process** |
| --- | --- | --- |
| Mannheim | DPO, REC | REC review 1 🡺 Approval from DPO 🡺 Response 1 🡺 Approval by REC |
| Thessaloniki | REC | REC review 🡺 Approval by REC |
| Brescia | REC | REC review 1 🡺 Response 1 🡺 REC review 2 🡺 data privacy impact assessment required (DPO) 🡺 Response 2 🡺 REC review 3 🡺 Representative of University of Oxford (data platform location) needed 🡺 Approval by REC |
| Stavanger | DPO, REC | REC review 1 🡺 Response 1 🡺 REC review 2 🡺 Approval of IT department 🡺 Approval of DPO 🡺 Approval by REC |
| Lisbon | REC | REC review 1 🡺 Response 1 🡺 Approval by REC |
| Barcelona | REC | REC review 1 🡺 Response 1 🡺 Approval by REC |
| Stockholm | REC | REC review 1 🡺 Response 1 🡺 REC review 2 🡺 Study rejected 🡺 Re-submission after adjustment of protocol 🡺 REC review 3 🡺 Response 3 🡺 Approval by REC |
| Amsterdam | Scientific board, REC, DPO, IT department | Scientific board review 1 🡺 Response 1 🡺 Approval scientific board 🡺 REC review 1 🡺 Response 1 🡺 REC review 2 🡺 Approval from DPO 🡺 Approval from IT 🡺 Response 2 🡺 REC review 3 🡺 Response 3 🡺Approval by REC 🡺 Approval by board of directors hospital |
| Oxford/London | REC | Approval by study sponsor, including approval by DPO 🡺 REC review 1 🡺 Response 1 🡺 Approval by REC 🡺 Approval of R&D departments of individual sites to ensure deliverability on a local level |

*Note. The second column shows the documents that are reviewed and coded during the study. The third column shows the review process, as visualized in Figure 1. Abbreviations: DPO = data privacy officer, REC = research ethics committee, R&D = research & development.*
