# Supplementary material for: Ethical challenges of using remote monitoring technologies for clinical research: A case study of the role of local research ethics committees in the RADAR-AD study
Source: PLoS One. 2023 Jul 7;18(7):e0285807. doi: 10.1371/journal.pone.0285807 (PMC10328231; doi:10.1371/journal.pone.0285807)
Supplement: Attachment — Submitted filename: PLOSONE_Rebuttal2_RADARAD_REC_20230421.docx [file pone.0285807.s003.docx]

Dear editor,

We would like to thank reviewer 1 and 3 for their additional comments on our manuscript entitled ‘Ethical challenges of using remote monitoring technologies for clinical research: a case study of the role of local Research Ethics Committees in the RADAR-AD study’. The comments were relevant and enabled us to improve the quality of our manuscript. Below, please find a point by point response to the provided comments:

**Reviewer #1: I thank the authors for addressing my previous concerns and suggestions. I have noticed 2 minor issues that should be addressed before the manuscript is ready for publication:**

1. The table in the supplementary material (line 514) is currently missing the content for the column entitled “documents reviewed”.

We have changed the header of the table and the title of the column to make it more clear what the column content entails.

2. Table 2 (line 141) needs a brief in-text explanation of what is meant by site-specific, region-specific, and country-specific.

We have added an in-text sentence (line 130-132), and also added a footnote to Table 2 (lines 145-147) to clarify what is meant by site-specific, region-specific, and country-specific.

**Reviewer #2: (No Response)**

**Reviewer #3: The revision has now addressed the comments of reviewers and provided explanations for the comments they disagreed with.**

1. In the responses, the authors gave a good explanation of the study design's inclusion of pre-clinical AD. They agreed that the study design "does not concern an issue with RMTs, but with the view of Alzheimer’s disease in general. It is however an example of how the view of RECs differs between countries. Importantly, the issues raised by several REC’s did concern the inclusion of a preclinical AD group within the context of RMT measurements, which does link to the goals of this study. Preclinical AD is a widely accepted study group in research, but is not used in clinical practice, as there is no treatment yet. In our view, RECs refusing to include preclinical AD in a clinical study are therefore withholding from an important step forwards in clinical trials for AD, and is therefore an important point to mention in the discussion of this manuscript." I believe this is a great point to include in the actual manuscript, particularly in the study description (lines 115-116) or in more detail in the discussion (308-312).

Thank you for this suggestion, we have added the explanation in the discussion (lines 313-314 and lines 319-322).

2. The list of citations includes a #61 that is blank.

Thank you for noticing, this 61 belongs however to the previous citation (#35) as part of the page numbers.

3. There are slight grammatical and spelling errors throughout. Please proofread. E.g., 'publically' line 119-120.

We have changed ‘publically’ to ‘publicly’ in line 119. We have proofread the manuscript and corrected grammatical and spelling errors.
